# Advanced aqueous rechargeable lithium battery using nanoparticulate LiTi_2_(PO_4_)_3_/C as a superior anode

**DOI:** 10.1038/srep10733

**Published:** 2015-06-02

**Authors:** Dan Sun, Yifan Jiang, Haiyan Wang, Yan Yao, Guoqing Xu, Kejian He, Suqin Liu, Yougen Tang, Younian Liu, Xiaobing Huang

**Affiliations:** 1 College of Chemistry and Chemical Engineering, Central South University, Changsha, 410083, P.R China; 2 Department of Electrical and Computer Engineering, University of Houston, Houston, TX 77204, USA; 3 Advanced Research Centre, Central South University, Changsha 410083, P.R. China; 4 College of Chemistry and Chemical Engineering, Hunan University of Arts and Science, Changde, 415000, P.R. China; 5 State Key Laboratory for Powder Metallurgy, Central South University, Changsha 410083, P.R. China

## Abstract

Poor cycling performance arising from the instability of anode is still a main challenge for aqueous rechargeable lithium batteries (ARLB). In the present work, a high performance LiTi_2_(PO_4_)_3_/C composite has been achieved by a novel and facile preparation method associated with an *in-situ* carbon coating approach. The LiTi_2_(PO_4_)_3_/C nanoparticles show high purity and the carbon layer is very uniform. When used as an anode material, the ARLB of LiTi_2_(PO_4_)_3_/C//LiMn_2_O_4_ delivered superior cycling stability with a capacity retention of 90% after 300 cycles at 30 mA g^−1^ and 84% at 150 mA g^−1^ over 1300 cycles. It also demonstrated excellent rate capability with reversible discharge capacities of 115 and 89 mAh g^−1^ (based on the mass of anode) at 15 and 1500 mA g^−1^, respectively. The superior electrochemical properties should be mainly ascribed to the high performance of LiTi_2_(PO_4_)_3_/C anode, benefiting from its nanostructure, high-quality carbon coating, appropriate crystal structure and excellent electrode surface stability as verified by Raman spectra, electrochemical impedance spectroscopy (EIS), X-ray diffraction (XRD) and scanning electron microscopy (SEM) measurements.

Concerns over global energy crisis and environmental pollution have spurred intensive researches on energy storage technologies to utilize renewable energy sources such as solar and wind^1^,^2^. Lithium ion batteries (LIBs) have been widely applied as the power sources for portable electronic devices and also have received overwhelming attention for electric vehicles (EVs) and large-scale energy storage system (ESS)^3^. However, high cost and safety issues arising from the usage of flammable organic electrolytes greatly limit its further applications. As a result, new energy storage systems with low cost and high reliability are urgently needed^4^. By using inexpensive salt solution as electrolyte, aqueous rechargeable lithium battery (ARLB) can fundamentally settle the safety issues and also avoid rigorous assembly conditions. Moreover, ARLB is far more environmental friendly compared with non-aqueous LIBs and the ionic conductivity of electrolyte can be increased by several magnitudes^5^.

However, poor cycling performance is still a big challenge for ARLB since Li ion intercalation processes in aqueous electrolyte are more complicated compared to those in organic electrolyte probably due to the side reaction of water^6^. Taking into account of hydrogen or oxygen evolution reaction, the choices of available electrode materials, anode materials in particular, are largely limited. Within the stable electrochemical window of water, the commercial cathode materials in LIBs including LiNi_1/3_Co_1/3_Mn_1/3_O_2_^7^, LiCoO_2_^8^,^9^,^10^, LiMn_2_O_4_^11^ and LiFePO_4_^12^ can be reversibly cycled in aqueous electrolyte and have been well studied as the cathodes for ARLB. As reported by Wu *et al.*^13^, porous LiMn_2_O_4_ nanograins showed a high capacity retention of 93% after 10000 cycles at a rate of 9C. The anode for ARLB requires the electrode material with an intercalation potential of 2~3 V *vs.* Li^+^/Li^14^. In this regard, there are only several kinds of suitable candidates. The first ARLB of VO_2_//LiMn_2_O_4_ was reported in 1994^15^, whose cycling stability was very poor. Since then, ARLBs of LiV_3_O_8_//LiMn_2_O_4_, LiV_3_O_8_//LiNi_0.81_Co_0.19_O_2_, NaV_3_O_8_//LiMn_2_O_4_, NaV_6_O_15_//LiMn_2_O_4_ and so on were constructed using vanadates as anodes^16^,^17^,^18^,^19^. However, most of these vanadates only delivered limited cycling life due to the materials dissolution in aqueous solution, especially at a low current density^6^. Until recently, LiTi_2_(PO_4_)_3_/C has shown the prospect as an anode for ARLB with high power density and long cycling life. In Wessells’s work^20^, LiTi_2_(PO_4_)_3_ exhibited a capacity retention of 89% even at a low current density of C/5 rate after 100 cycles in aqueous electrolyte. By eliminating the soluble oxygen in Li_2_SO_4_ solution, the cycling life of LiTi_2_(PO_4_)_3_//LiFePO_4_ ARLB constructed by Xia *et al.*^21^ was up to 1000 cycles at a current rate of 6C. Unfortunately, the cycling stability of such ARLB system at a low current density was still insufficient (85% after 50 cycles at a current rate of 8 hrs for a full charge/discharge test). Further efforts should be carried out to continue improving the electrochemical stability for ARLB.

LiTi_2_(PO_4_)_3_ (LTP) reacts electrochemically with lithium at 2.5 V *vs.* Li^+^/Li for Ti^4+^/Ti^3+^ couple and Li ions occupy the octahedral interstitial sites representing M(1) within the LTP structure (space group *R*3*c*)^22^. Although LTP possesses excellent operating potential, flat voltage plateau as well as relatively high chemical stability in aqueous electrolytes, the conductivity of pure LTP has been found to be relatively low for practical use^23^. Size miniaturization and carbon coating are simple and effective ways to address such issue. As we know, synthetic strategies, coating strategies and carbon sources can greatly affect the electrochemical performance. In most reports, LTP/C was synthesized by solid state reaction^24^,^25^,^26^ and Pechini method^20^ followed by a subsequent carbon coating. Generally, the common synthetic methods need a high sintering temperature and a long annealing time, resulting in severe aggregation of the particles. Furthermore, the two-step carbon coating process, in which the carbon source was mixed with the precursor mechanically, often leads to heterogeneous coating. Thus new preparation and carbon coating strategies should be designed to achieve high performance LTP/C.

Hydrolysis method has been widely applied for preparing electrode materials due to its special virtues^27^. It could provide a uniform mixture of raw materials at molecular level and achieve the controlling of the particle size *via* hydrolysis rate. It is well accepted that *in-situ* carbon coating tends to produce a homogeneous and tight carbon layer on the surface of particles, which is vital to the improvement of electrochemical performance^28^,^29^,^30^. Accordingly, a novel and facile hydrolysis method associated with *in-situ* carbon coating was developed for LTP/C in this work. This method is of time-saving, easy to operate and also allows for mass-scale production, which are primary concerns for commercial applications of LIBs. The selected carbon sources can directly affect the characteristics of the carbon additive, in terms of its structure, distribution and thickness of carbon coating layer, which are proportional to the performance of carbon coated composite electrode^28^,^30^. In this view, the carbon sources were also optimized to obtain high-quality carbon layer. Combining these methods, high performance LTP/C has been achieved in the present study. To the best of our knowledge, the reported LTP/C composite exhibited the longest cycling stability in aqueous electrolyte at relatively low current densities (e.g. 1 C) when used as an anode for ARLB. It also demonstrated excellent high-rate capability.

## Results

**Figure 1:**
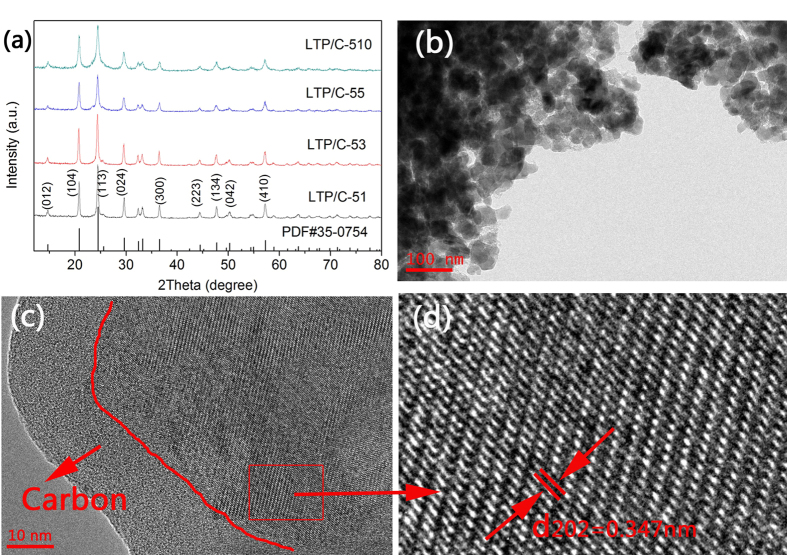
(**a**) XRD patterns of as-prepared LTP/C composites; (**b**) TEM and (**c**) HRTEM images of as-prepared LTP/C-55; (**d**) Enlarged view of the red box in (c) showing the lattice fringes of LTP.

The XRD patterns of as-prepared LTP/C composites with various carbon contents are presented in Fig. 1a, from which it can be seen that all samples demonstrate similar diffraction patterns, which can be well indexed to LiTi_2_(PO_4_)_3_ phase with a rhombohedral NASICON type structure and a *R*3*c* space group (JCPDS#35-0754). The absence of impurity peak implies the high purity of LTP phase in the samples. The measured lattice parameters of as-prepared samples in Table S1 are all in good agreement with those reported results^22^,^26^. The microstructural features of as-prepared LTP/C-55 composite are shown in Fig. 1(b,c). As displayed, LTP/C-55 is composed of individual particles with the average size of less than 80 nm and slight agglomeration takes place. The Brunauer-Emmet-Teller (BET) surface area of LTP/C-55 (Fig. S1, see the Supporting Information) is 50.620 m^2^ g^−1^, much larger than that prepared by solid state reaction^26^. It is well known that a larger surface area of electrode material will result in a shorter lithium ion diffusion path and enough contact between the active material and electrolyte, probably leading to higher electrochemical performance for ARLB. The HRTEM image (Fig. 1c) clearly reveals the presence of an amorphous carbon layer on the surface of the LTP particles. This carbon layer is very uniform along the whole particle surface and the thickness is less than 20 nm. This well-distributed carbon layer would ensure the electrode material transfer electrons along all directions during charge and discharge processes^31^. This is much better than the conventional coating method like coprecipitation and ball-milling methods^31^. There is no doubt that the uniform carbon layer is ascribed to the *in-situ* coating strategy, in which the carbon source, phenolic resin could be dissolved in ethanol and well dispersed on the surface of precursor. The high-quality carbon layer could provide a careful protection for inner electrode and thus a high electrical conductivity could be achieved. An enlarged view of the lattice fringes is presented in Fig. 1d and the inter-planar spacing deduced from the image is ~0.347 nm, which corresponds to the d-spacing of the (202) plane of rhombohedral LTP. The carbon contents of LTP/C composites are measured by TGA. As recorded in Fig. S2 (a,b,c and d), the amount of coated carbon for LTP/C-51, LTP/C-53, LTP/C-55 and LTP/C-510 are 1.9 wt%, 4.5 wt%, 6.2 wt% and 13.4 wt%, respectively.

**Figure 2:**
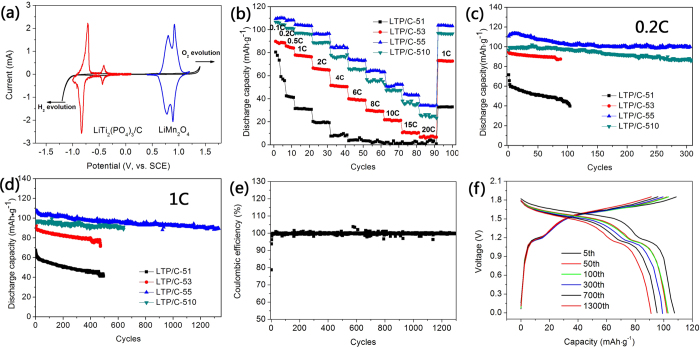
(**a**) Cyclic voltammetry (CV) curves of as-prepared LTP/C-55 and LiMn_2_O_4_ electrode in solution Li_2_SO_4_ solution at a sweep rate of 0.4 mV s^−1^, respectively, measured by a three-electrodes system using a platinum sheet as the counter electrode and a saturated calomel electrode (SCE) as the reference electrode. (**b**) Discharge capacities of LTP/C//LiMn_2_O_4_ ARLB at various rates. (**c**)-(**d**) Cycling performance of LTP/C//LiMn_2_O_4_ ARLB at 0.2C and 1C, respectively. (**e**) Coulombic efficiency of LTP/C-55//LiMn_2_O_4_ ARLB at 1C. (**f**) Discharge curves of LTP/C-55//LiMn_2_O_4_ ALRB at different cycles at 1C. The capacity was based on the mass of LTP/C composite in this paper.

Li ion intercalation/deintercalation behaviors of LTP/C-55 and LiMn_2_O_4_ electrodes in aqueous electrolyte were investigated by three-electrode CV measurement (Fig. 2a), respectively. Clearly, LTP/C-55 demonstrates two reduction peaks (~−0.83 V and −0.44 V, respectively) between 0 and −1.0 V *vs.* SCE. And the corresponding oxidation peaks are located at ~−0.73 V and −0.42 V *vs.* SCE. Excellent kinetics behaviors imply that LTP/C could be used as a promising anode for ARLB. Not all cathode materials possess the best stability in neutral electrolyte, for example, LiNi_1/3_Co_1/3_Mn_1/3_O_2_ is more stable in Li_2_SO_4_ solution with pH = 11 due to less H^+^ co-intercalation^7^. LiFePO_4_ and LiMn_2_O_4_ could cycle stably in neutral aqueous electrolyte^21^,^22^. Accordingly, in view of its relatively high intercalated potential, low cost and excellent cycling stability in lithium-containing solution, commercial LiMn_2_O_4_ was directly used in the present work as the cathode. Good lithium insertion/extraction behavior is also demonstrated in Fig. 2a. The typical CV curves of LTP/C//LiMn_2_O_4_ ARLB are compared in Fig. S3. As can be seen, LTP/C-55//LiMn_2_O_4_ exhibits the best reversibility with two main oxidation peaks locating at *ca.* 1.21 V and 1.78 V, respectively and the corresponding reduction peaks at *ca.* 1.12 V and 1.54 V, respectively. No obvious peaks corresponding to the evolution of hydrogen and oxygen are observed, which is consistent with the high Coulumbic efficiency (>99%) in Fig. 2e. It is noted that the polarization potential (ΔE) decreases firstly and then increases with the increased carbon content. The increase of carbon content is generally beneficial for the improvement of the conductivity as well as the thickness of carbon layer^32^. The improved conductivity could suppress the electrode polarization while a thick inert carbon layer would conversely restrict both the penetration of electrolyte and the transfer of Li ions. Apparently, LTP/C-55 has achieved a good balance between the conductivity and Li ions transfer^31^. The rate performance of LTP/C with different carbon contents are depicted in Fig. 2b. It can be clearly seen that the LTP/C-55 exhibits the best rate performance with a discharge capacity of 110, 104.4, 96.2, 84.7, 74.8, 63.5 and 57.8 mAh g^−1^ (based on the mass of LTP/C) at 0.1C, 1C, 2C, 4C, 6C, 8C and 10C (1C = 150 mA g^−1^), respectively. Considering there is a 6.2 wt% carbon in the composite, the real capacity calculated from the bare LTP is 117.3 mAh g^−1^ at 0.1C, about 85% of the theoretical value (138 mAh g^−1^). The rate performance is gradually improved with the increase of carbon content due to the enhanced electronic conductivity. However, LTP/C-510 with too much carbon delivers a much lower reversible discharge capacity in comparison with LTP/C-55, which is in good agreement with CV results. Fig. 2(c,d) shows the cycling performance of as-prepared LTP/C samples at 0.2C and 1C, respectively. Generally, the cycling performances of LTP/C-55 and LTP-510 at both 0.2C and 1C are superior to LTP/C-51 and LTP/C-53, and the cycling stability increases as the carbon content increases. The carbon coating layer could function as a multi-purpose layer between the active electrode and electrolyte to enhance the electrode conductivity, suppress water splitting, protect the active material from electrolyte corrosion, and maintain the electrode integration and conductivity upon volume change, thus resulting in much improved rate capability and cycling stability for the coated materials^33^. The poor electrochemical properties of bare LTP could well prove it (see Fig. S4). An appropriate carbon layer could improve the electrochemical properties of electrode to the greatest extent with the lowest sacrifice of reversible capacity. As a result, LTP/C-55 shows the best electrochemical properties due to its optimum carbon content. As Fig. 2c shows, at a low rate of 0.2C, it delivers a discharge capacity of 110.6 mAh g^−1^, and 102.5 mAh g^−1^ is maintained after 300 cycles with a capacity retention of 90%. At 1C, a discharge capacity of 106 mAh g^−1^ is demonstrated and 84% of the initial discharge capacity is kept after 1300 cycles. To our best knowledge, the cycling performance at the relatively low current density (1C) is much superior to those of all those reported vanadium oxides or vanadates and LTP/C as anode materials to date (see Table S2), such as Na_0.33_V_2_O_5_^34^, Na_2_V_6_O_16_·xH_2_O^18^, VO_2_(B)^15^, LiV_3_O_8_^8^,^35^ and LTP/C^26^. VO_2_(B)//LiMn_2_O_4_ reported by Dahn^15^ can only be cycled for 25 cycles. LiV_3_O_8_//LiMn_2_O_4_ showed 53.5% of the initial capacity after 100 cycles^17^. Na_2_V_6_O_16_·0.14H_2_O//LiMn_2_O_4_ and NaV_6_O_15_//LiMn_2_O_4_ with capacity retention of 77% after 200 cycles and 80% after 400 cycles were demonstrated in our previous work^18^,^19^. Carbon coated LTP delivered a discharge capacity of 113 mAh g^−1^ at 0.2C^6^ and maintained 89% of the initial capacity after 100 cycles. LTP//LiMn_2_O_4_ reported by Xia *et al.* exhibited a capacity retention of 82% after 200 cycles at a current rate of 10 mA cm^−2^. It is worthy noting that, in subsequent their work, the capacity retention of LTP/C//LiFePO_4_ ARLB was over 90% after 1000 cycles when fully charged/discharged in 10 min *via* eliminating the soluble oxygen in electrolyte^21^. However, at a low current rate of 8 hours, the capacity retention was only 85% after 50 cycles, probably due to the instability of LTP/C anode.

The decomposition of water^14^ and the interaction between aqueous electrolyte and electrode surface^16^, often result in relatively low Coulombic efficiency^25^, which are considered as the important origins of capacity fading for ARLB, particularly at low current densities. As shown in Fig. 2e, the overall average Coulombic efficiency of LTP/C-55//LiMn_2_O_4_ in this work is >99%, except for the initial cycles. Fig. 2f presents the corresponding discharge curves of LTP/C-55//LiMn_2_O_4_ ALRB after different cycles. The discharge plateaus around 1.55 V and 1.0 V agree well with the CV results. After 1300 cycles, the plateau still remains well-shaped, suggesting superior crystal stability for both the anode and cathode. The capacity decay is mainly attributed to the slight shrinkage of the plateau around 1.55 V.

**Figure 3:**
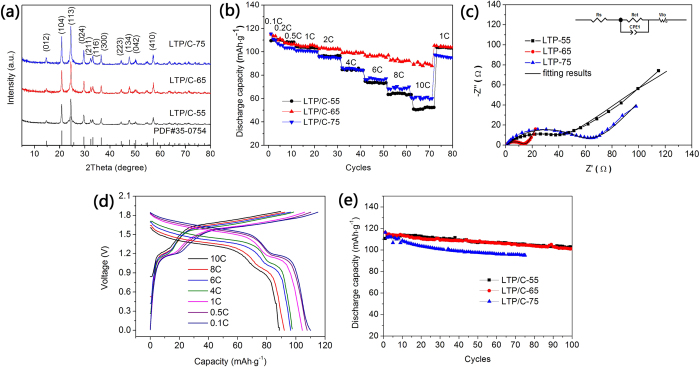
(**a**) XRD patterns of as-prepared LTP/C with different calcination time.(**b**) Rate performance of LTP/C//LiMn_2_O_4_ ARLB from 0.1C to 10C. (**c**) EIS results of LTP/C//LiMn_2_O_4_ ARLB, and the equivalent circuit model (inset). Before testing, each cell was cycled for 5 times at 1C. (**d**) Charge/discharge curves of LTP/C-65//LiMn_2_O_4_ at various rates from 0.1C to 10C. (**e**) Cycling performance of LTP/C//LiMn_2_O_4_ ARLB at 1C.

Further investigation implies that the calcination time could have a strong effect on the electrochemical properties^31^. Figure 3a shows the XRD patterns of LTP/C sintered at 700 °C for different period of time. All samples present similar diffraction peaks and are in good match with the standard LTP PDF card (JCPDS#35-0754). High phase purity is also observed. The calculated lattice parameters of as-prepared samples are listed in Table S3. Of these samples, interestingly, the sample sintered for 6 hrs (LTP/C-65) has the largest crystal volume. The rate performance of as-prepared LTP/C samples can be compared in Fig. 3b. LTP/C-65 shows the best rate capability with a discharge capacity of 115 mAh g^−1^ at 0.1C. When the rate increases to 10C, a discharge capacity of 89.0 mAh g^−1^ is still maintained based on the whole mass of LTP/C. To our best knowledge, no study so far has achieved such good rate performance for LTP in aqueous electrolyte. The improved rate performance of LTP/C-65 compared with other samples may have a correlation with its largest crystal volume as calculated in Table S3. Generally, larger crystal volume will afford more comfortable diffusion pathway for Li ion and thus allow a faster diffusion. EIS results of LTP/C with different sintering time in Fig. 3c could well support this statement. The plots consist of a depressed semicircle in the high frequency regions and a straight line in the low frequency region. The semicircle at high frequency can be assigned to the charge-transfer impedance (R_ct_) on electrode-electrolyte interface, whereas the line region corresponds to the Warburg impedance, which reflects Li ion diffusion in the solid state electrodes^36^,^37^. For comparison, LTP/C-65 shows the smallest R_ct_ value (12 Ω, see Table S4), which is consistent with the best rate capability in Fig. 3b. As measured by the four-point probe method, LTP/C-65 also shows the highest electronic conductivity (5.4 × 10^−4^ s/cm), closed to three times of that of LTP/C-55 (2.0 × 10^−4^ s/cm), further implying the effect of the calcination time. Fig. 3d gives the corresponding charge and discharge curves of LTP/C-65 at different rates. A long voltage plateau around 1.55 V and a short plateau around 1.0 V are observed at low rate. On increasing the current density, good plateaus are still maintained, though the increased polarization potential is displayed. The effect of calcination time on cycling stability can be further investigated in Fig. 3e. Clearly, LTP/C-65 exhibits very closed cycling performance to LTP/C-55, while LTP/C-75 shows a much inferior one. That is, a good balance between cycling stability and rate capability has been achieved for LTP/C-65.

## Discussion

As mentioned in the introduction, one of the important determining factor for the quality of carbon coating layer is the carbon source. To reveal the merits of phenonic resin, sucrose was also employed as the carbon source for comparison. The XRD pattern of as-prepared LTP/C sample is demonstrated in Fig. S5a. A relatively pure phase of LTP with the space group of *R*3*c* is shown. According to TEM images (Fig. S5(b,c)), the LTP/C using sucrose as the carbon source is composed of nanoparticles, whose size is less than 100 nm. However, severe particle agglomeration happens. An amorphous carbon layer is also illustrated by HRTEM images (Fig. S5(d,e)). Fig. S5f demonstrates a carbon content of 7.8 wt% for LTP/C using 0.5 g of sucrose as the carbon source. Thus, for accuracy, the electrochemical properties of LTP/C using 0.5 g of phenolic resin and 0.5 g of sucrose as the carbon sources are compared in details.

Cycling performance of LTP/C//LiMn_2_O_4_ ARLB at 1C using different carbon sources is shown in Fig. S6a. For simplicity, LTP/C using phenolic resin and sucrose as the carbon sources is denoted as LTP/C-RF and LTP/C-SR, respectively. It is clearly that, although the discharge capacity of LTP/C-SR is higher than that of LTP/C-RF, it fades sharply in the first 20 cycles. The discharge capacity at high rates (Fig. S6b) further manifests the inferior rate property of LTP/C-SR. Note that after deep cycling at 10C, a constant capacity of around 110 mAh g^−1^ can be restored at 1C for LTP/C-RF, in contrast only 15 mAh g^−1^ for LTP/C-SR. To find out the reasons, Raman spectra of LTP/C composites using different carbon sources were performed and the results are shown in Fig. S6c. The band in the range of 1150–1450 cm^−1^ (centered on 1330 cm^−1^) is attributed to the D-band of carbon, which is indicative of the sp^2^ disordered induced phonon mode, whereas that centered on 1605 cm^−1^ is due to the G-band (sp^2^ graphite band)^30^,^38^. It was well verified that the structure of the carbon, particularly the sp^2^/sp^3^ character, can strongly influence the electronic conductivity^28^. Generally, the electrode materials containing more graphitic carbon i.e., those with higher sp^2^/sp^3^, can outperform those containing larger amounts of a less conductive coating layer^28^,^31^. Accordingly, the intensity ratios of the D band to the G band for LTP/C-SR and LTP/C-RF are estimated to be about 0.94 and 0.85, respectively. The lower D/G ratio of carbon for LTP/C-RF implies a higher electronic conductivity than LTP/C-SR. EIS results (Fig. S6d) are also measured. As displayed, the plots consist of a depressed semicircle which represents the R_*ct*_ in the high frequency regions and a straight line which could be assigned to Warburg impedance in the low frequency region. Obviously, the R_ct_ value of LTP/C-RF electrode is much smaller than that of LTP/C-SR, confirming the higher conductivity of LTP/C-PF. The related results provide clear evidence for the merit of phenolic resin as the carbon source for producing the high-order and uniform carbon coated layer^29^,^31^.

**Figure 4:**
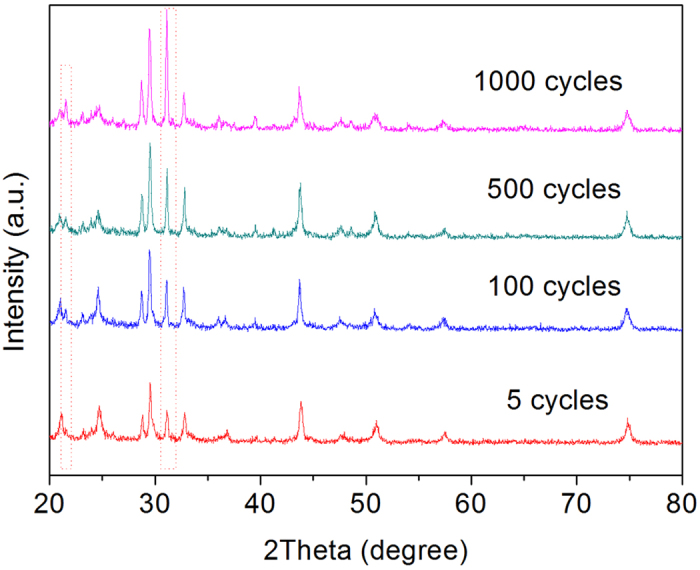
XRD patterns of LTP/C-55 electrodes after different cycles at 1C. Before disassembling, each cell was charged to 1.6 V and then kept at that voltage for 2 hrs.

It has been suggested that the capacity fading of ARLB could be related to transition metal ion dissolution, phase transformation of electrode material, decomposition of water, and electrode surface corrosion by water^14^. Wang *et al.*^39^ confirmed that the crystalline structure of Li_x_V_2_O_5_ became nearly amorphous after 40 cycles in ARLB. The formation of new compounds was also considered to be the cause for capacity fading of TiP_2_O_7_ by Chen and his group^25^. It is easy to assume that the high-quality and full carbon coated layer could protect active material from electrolyte corrosion, and maintain the electrode crystal structure, integration and conductivity upon volume change resulting better cycling stability. To prove this statement, XRD and SEM measurements are conducted for further analysis. Fig. 4 shows the XRD patterns of LTP/C-55 electrodes after different cycles (5, 100, 500, 1000 cycles). Apart from the intensity change in some diffraction peaks, which is probably due to the smooth surface of the electrode film^40^, all the XRD patterns of electrodes are similar to those of LTP/C powder in Fig. 1a. As the cycle process proceeds, the intensity of diffraction peaks located at 2θ = 21.61° and 31.23° is increased remarkably, indicating a structure rearrangement. Note that the patterns of LTP/C-55 electrodes after different cycles show no degradation or new impurity peaks when compared with that after 5 cycles, implying the excellent structure stability of LTP/C-55 anode. Superior cycling stability has been also observed for LiMn_2_O_4_ cathode in corresponding XRD patterns after different cycles (Fig. S7). As reported by Wu *et al.*^13^, LiMn_2_O_4_ in aqueous electrolyte using activated carbon as the counter electrode could be well cycled even up to 10000 cycles. That is, LiMn_2_O_4_ could be well cycled in ARLB because of the absence of HF in aqueous solution. In the present work, the high-performance LTP/C anode should be a crucial reason for the superior electrochemical properties.

**Figure 5:**
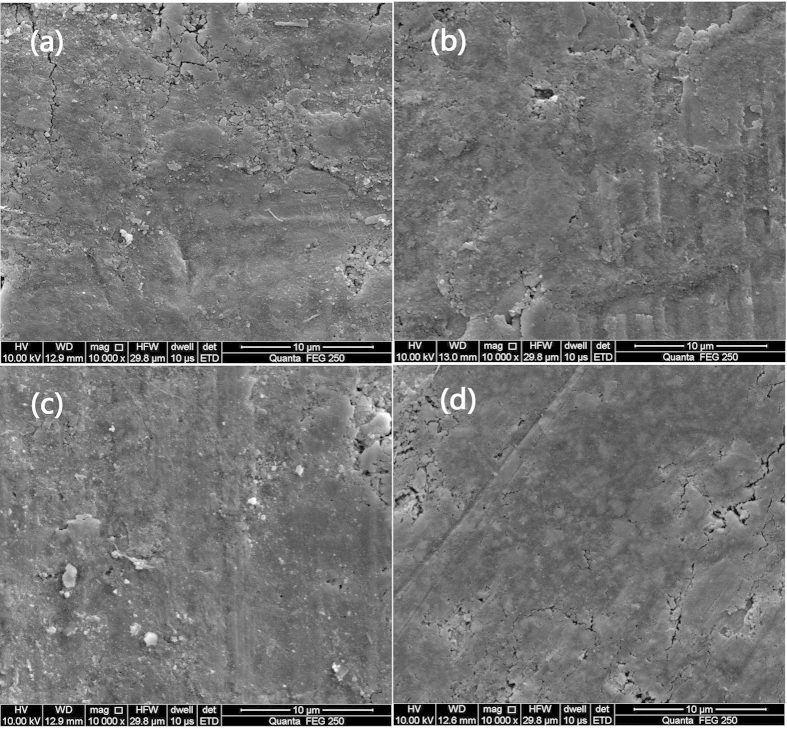
SEM images of LTP/C electrodes after different cycles: 5 cycles (**a**), 100 cycles (**b**), 500 cycles (**c**) and 1000 cycles (**d**).

Since the electrode is soaked in aqueous solution, the uninterrupted attacking by H_2_O would probably result in the dissolution of surface active materials and thus further damage the integrity of electrode surface. Perfect surface coating is considered as an effective approach to address such issue^14^. Fig. 5 shows surface microstructural features of LTP/C-55 electrode after 5, 100, 500 and 1000 cycles at 1C rate. As displayed, the electrode surface of LTP/C-55 after 1000 cycles still remains well in comparison with the electrode after 5 cycles. The mild damage of electrode surface, which corresponds well with the slight capacity fading, suggests a relatively stable electrode surface and effective suppression of the dissolution of LTP/C-55 electrode. Similar results have been also reported in Ref. 41,42. XRD and SEM results reveal that the stable crystal structure and electrode surface of LTP/C-55 thanks to the protection of full and high-quality carbon layer by *in-situ* coating approach should be the main reasons for superior cycling performance.

In summary, high-purity LTP/C nanoparticles with a homogeneous amorphous carbon layer were synthesized using phenolic resin as the carbon source by an *in-situ* coating approach. When used as an anode for ARLB, the optimized LTP/C composite electrode showed superior cycling stability with a capacity retention of 84% after 1300 cycles at 150 mA g^−1^. A high discharge capacity of 89.0 mAh g^−1^ based on the mass of LTP/C was also observed even at a current density of 1500 mA g^−1^, indicating excellent rate capability. It is believed that the hydrolysis method associated with *in-situ* coating approach played an important role for such superior electrochemical properties, by which nano-sized LTP/C composite with high phase purity and full carbon coating has been achieved. Moreover, the high-quality carbon coating layer carbonized from phenolic resin greatly contributes to the observed superior electrochemical properties. This work could provide effective strategies for preparation of other high-performance LiFePO_4_, LiMnPO_4_, Li_3_V_2_(PO_4_)_3_ and so on.

## Methods

### Synthesis of LTP/C composite

Firstly, 1.7570 g of phosphoric acid was dissolved in ethanol for the standby application. 3.3453 g of tetrabutyl titanate, 0.5134 g of lithium acetate and a certain amount carbon source (phenolic resin, provided by BTR Battery Materials Co., Ltd) were dissolved in ethanol with stirring. Then H_3_PO_4_/ethanol solution was dropwise added into the mixed solution. Afterwards, the mixed solution was refluxed at 55 °C for 3 hrs. Then the reflux system was removed and the temperature was increased to 80 °C to evaporate the solvent. The resulting precursor was finely ground by agate mortar and then pressed into pellets and calcined at 700 °C for a certain period of time under a mixed flowing H_2_/Ar (5:95 by volume).

To optimize the carbon content, the amount of added phenolic resin were 0.0, 0.1, 0.3, 0.5 and 1.0 g, respectively (the as-prepared samples were denoted as LTP, LTP/C-51, LTP/C-53, LTP/C-55 and LTP/C-510, respectively), and the calcination time of samples was 5 hrs. For comparison, LTP/C using 0.5 g of sucrose as the carbon source was also prepared in a similar way. The effect of calcination time on LTP was also investigated. According to the result, the sample with 0.5 g of phenolic resin possessed the best electrochemical properties, so we optimized the calcination time (5 hrs, 6 hrs and 7 hrs, denoted as LTP/C-55, LTP/C-65 and LTP/C-75, respectively) to further improve the rate performance of LTP.

### Characterizations

All X-ray diffraction (XRD) data were obtained by X-ray diffractometer (DX-2700, Dandong Haoyuan) utilizing a Cu-Kα1 source with a step of 0.02°. Note that XRD measurement of electrodes was different from that of LTP/C powder. The whole electrode consisting of active material, Super P carbon and polytetrafluoroethylene (PTFE), after washing with distilled water and drying for several hours, was directly used to perform the XRD test. No signal of stainless steel mesh was observed probably due to the thick electrode film, as reported in our previous work^19^. Before disassembling, each cell was charged to 1.6 V and kept at that voltage for more than 2 hrs. Microstructural studies of electrodes after different cycles were conducted using a scanning electron microscope (FEI Quanta 250 FEG, FEI Inc.). TEM and high resolution TEM (HRTEM) images of as-prepared LTP/C powder were obtained using JEOL JEM-2100F TEM with a LaB_6_ filament as the electron source. Brunauer-Emmet-Teller (BET) surface area of the samples was detected by nitrogen adsorption/desorption at -196 °C using a Builder SSA-4200 apparatus. Raman spectra were investigated with LabRAM Aramis (HORIBA Jobin Yvon) spectrometer. The electronic conductivity was measured by the four-point probe method (Guangzhou 4 Probes Tech, RTS-9). Thermogravimetric analysis (TGA) was performed on a STA 449C with a heating rate of 10 °C/min from 25 to 800 °C.

### Electrochemical measurements

The used LiMn_2_O_4_ was provided by Hunan Reshine New Material Co., Ltd. The LTP/C and LiMn_2_O_4_ electrodes were made in a similar way. Tested electrodes were obtained by pressing a mixture of active material, Super P carbon and PTFE in a weight ratio of 80:10:10 using distilled water as solvent on a stainless steel mesh and then dried at 110 °C for 8 hrs. Cyclic voltammetry (CV) of LTP/C and LiMn_2_O_4_ electrodes was performed using a three electrode system, where the tested electrode was used as the working electrode, platinum sheet electrode and saturated calomel electrode (SCE, 0.242 V *vs.* SHE: standard hydrogen electrode) as the counter and reference electrodes, respectively. CV test was carried out at room temperature using an electrochemical station (CHI660D). CR2016 coin-type cells were constructed by using LiMn_2_O_4_ electrode as the cathode, LTP/C electrode as the anode, and Li_2_SO_4_ (2 mol L^−1^) as the electrolyte. To evaluate the electrochemical properties of LTP/C exactly, an appropriate excessive LiMn_2_O_4_ was designed. The mass load density of LTP/C electrode was 3~4 mg/cm^2^ and the mass ratio of LiMn_2_O_4_ to LTP/C was ~1.5. The Li_2_SO_4_ electrolyte was pre-treated by flowing argon injection into the solution to eliminate the soluble oxygen. Charge and discharge tests were conducted under a desired current density by a Neware battery testing system (CT-3008W) at room temperature. Electrochemical impedance spectroscopy (EIS) was recorded by a Princeton workstation (PARSTAT2273, EG&G, US) over the frequency range from 100 kHz to 10 mHz with an amplitude of 5 mV. Before testing, the measured cell was charged to 1.6 V at 150 mA g^−1^, and then held for 2 hrs to reach a stable state.

## Additional Information

**How to cite this article**: Sun, D. *et al.* Advanced aqueous rechargeable lithium battery using nanoparticulate LiTi_2_(PO_4_)_3_/C as a superior anode. *Sci. Rep.* **5**, 10733; doi: 10.1038/srep10733 (2015).

## Supplementary Material

Supplementary Information

## References

[b1] Chiang Y. M. Building a better battery. Science 330, 1485–1486 (2010). 21148377 10.1126/science.1198591

[b2] Li Z., Young D., Xiang K., Carter W. C. & Chiang Y. M. Towards high power high energy aqueous sodium-ion batteries: the NaTi_2_(PO_4_)_3_/Na_0. 44_MnO_2_ System. Adv. Energy Mater . 3, 290–294 (2013).

[b3] Armand M. & Tarascon J. M. Building better batteries. Nature 451, 652–657 (2008). 18256660 10.1038/451652a

[b4] Tang W. *et al.* Aqueous rechargeable lithium batteries as an energy storage system of superfast charging. Energy Environ. Sci. 6, 2093–2104 (2013).

[b5] Wang G. J. *et al.* Electrochemical intercalation of lithium ions into LiV_3_O_8_ in an aqueous electrolyte. J. Power Sources 189, 503–506 (2009).

[b6] Cui Y. *et al.* Synthesis andelectrochemical behavior of LiTi_2_(PO_4_)_3_ as anode materials for aqueous rechargeable lithium batteries. J. Electrochem. Soc. 160, A53–A59 (2013).

[b7] Wang Y. G., Luo J. Y., Wang C. X. & Xia Y. Y. Hybrid aqueous energy storage cells using activated carbon and lithium-ion intercalated compounds II. Comparison of LiMn_2_O_4_, LiCo_1∕3_Ni_1∕3_Mn_1∕3_O_2_, and LiCoO_2_ positive electrodes. J. Electrochem. Soc. 153, A1425–A1431 (2006).

[b8] Tang W. *et al.* Nano-LiCoO_2_ as cathode material of large capacity and high rate capability for aqueous rechargeable lithium batteries. Electrochem. Commun. 12, 1524–1526 (2010).

[b9] Wang G. J. *et al.* An aqueous rechargeable lithium battery based on doping and intercalation mechanisms. J. Solid State Electrochem. 14, 865–869 (2010).

[b10] Ruffo R., La Mantia F., Wessells C., Huggins R. A. & Cui Y. Electrochemical characterization of LiCoO_2_ as rechargeable electrode in aqueous LiNO_3_ electrolyte. Solid State Ionics 192, 289–292 (2011).

[b11] Cui Y., Yuan Z., Bao W., Zhuang Q. & Sun Z. Investigation of lithium ion kinetics through LiMn_2_O_4_ electrode in aqueous Li_2_SO_4_ electrolyte. J. Appl. Electrochem. 42, 883–891 (2012).

[b12] He P., Liu J. L., Cui W. J., Luo J. Y. & Xia Y. Y. Investigation on capacity fading of LiFePO_4_ in aqueous electrolyte. Electrochim. Acta 56, 2351–2357 (2011).

[b13] Qu Q. *et al.* Porous LiMn_2_O_4_ as cathode material with high power and excellent cycling for aqueous rechargeable lithium batteries. Energy Environ. Sci. 4, 3985–3990 (2011).

[b14] Wang Y., Yi J. & Xia Y. Recent progress in aqueous lithium-ion batteries. Adv. Energy Mater . 2, 830–840 (2012).

[b15] Li W., Dahn J. R. & Wainwright D. S. Rechargeable lithium batteries with aqueous electrolytes. Science 264, 1115–1118 (1994). 17744893 10.1126/science.264.5162.1115

[b16] Zhao M., Zheng Q., Wang F., Dai W. & Song X. Electrochemical performance of high specific capacity of lithium-ion cell LiV_3_O_8_//LiMn_2_O_4_ with LiNO_3_ aqueous solution electrolyte. Electrochim. Acta 56, 3781–3784 (2011).

[b17] Wang G. J., Zhang H. P., Fu L. J., Wang B. & Wu Y. P. Aqueous rechargeable lithium battery (ARLB) based on LiV_3_O_8_ and LiMn_2_O_4_ with good cycling performance. Electrochem. Commun. 9, 1873–1876 (2007).

[b18] Zhou D., Liu S., Wang H. & Yan G. Na_2_V_6_O_16_·0.14H_2_O nanowires as a novel anode material for aqueous rechargeable lithium battery with good cycling performance. J. Power Sources 227, 111–117 (2013).

[b19] Sun D. *et al.* Aqueous rechargeable lithium batteries using NaV_6_O_15_ nanoflakes as high performance anodes. J. Mater. Chem. A 2, 12999–13005 (2014).

[b20] Wessells C., Huggins R. A. & Cui Y. Recent results on aqueous electrolyte cells. J. Power Sources 196, 2884–2888 (2011).

[b21] Luo J. Y., Cui W. J., He P. & Xia Y. Y. Raising the cycling stability of aqueous lithium-ion batteries by eliminating oxygen in the electrolyte. Nat. Chem . 2, 760–765 (2010). 20729897 10.1038/nchem.763

[b22] Shivashankaraiah R. B., Manjunatha H., Mahesh K. C., Suresh G. S. & Venkatesha T. V. Electrochemical characterization of LiTi_2_(PO_4_)_3_ as anode material for aqueous rechargeable lithium batteries. J. Electrochem. Soc. 159, A1074–A1082 (2012).

[b23] Nuspl G. *et al.* Lithium ion migration pathways in LiTi_2_(PO_4_)_3_ and related materials. J. Appl. Phys. 86, 5484–5491 (1999).

[b24] Liu X. H., Saito T., Doi T., Okada S. & Yamaki J. I. Electrochemical properties of rechargeable aqueous lithium ion batteries with an olivine-type cathode and a Nasicon-type anode. J. Power Sources 189, 706–710 (2009).

[b25] Wang H., Huang K., Zeng Y., Yang S. & Chen L. Electrochemical properties of TiP_2_O_7_ and LiTi_2_(PO_4_)_3_ as anode material for lithium ion battery with aqueous solution electrolyte. Electrochim. Acta 52, 3280–3285 (2007).

[b26] Luo J. Y. & Xia Y. Y. Aqueous lithium-ion battery LiTi_2_(PO_4_)_3_/LiMn_2_O_4_ with high power and energy densities as well as superior cycling stability. Adv. Funct. Mater. 17, 3877–3884 (2007).

[b27] Wang X. Y., Li Y. J., Xu C., Kong L. & Li L. Synthesis and characterization of Li_4_Ti_5_O_12_ via a hydrolysis process from TiCl_4_ aqueous solution. Rare Met . 33, 459–465 (2014).

[b28] Doeff M. M., Hu Y., McLarnon F. & Kostecki R. Effect of surface carbon structure on the electrochemical performance of LiFePO_4_. Electrochem. Solid State Lett. 6, A207–A209 (2003).

[b29] Sun D. *et al.* In-situ synthesis of carbon coated Li_2_MnSiO_4_ nanoparticles with high rate performance. J. Power Sources 242, 865–871 (2013).

[b30] Hu Y., Doeff M. M., Kostecki R. & Fiñones R. Electrochemical performance of sol-gel synthesized LiFePO_4_ in lithium batteries. J. Electrochem. Soc. 151, A1279–A1285 (2004).

[b31] Wang J. & Sun X. Understanding and recent development of carbon coating on LiFePO_4_ cathode materials for lithium-ion batteries. *Energy* Environ. Sci. 5, 5163–5185 (2012).

[b32] Cho Y. D., Fey G. & Kao H. M. The effect of carbon coating thickness on the capacity of LiFePO_4_/C composite cathodes. J. Power Sources 189, 256–262 (2009).

[b33] Li H. & Zhou H. Enhancing the performances of Li-ion batteries by carbon-coating: present and future. Chem. Commun. 48, 1201–1217 (2012). 10.1039/c1cc14764a 22125795

[b34] Xu Y., Han X., Zheng L., Yan W. & Xie Y. Pillar effect on cyclability enhancement for aqueous lithium ion batteries: a new material of β-vanadium bronze M_0.33_V_2_O_5_ (M=Ag, Na) nanowires. J. Mater. Chem. 21, 14466–14472 (2011).

[b35] Dubarry M. *et al.* Synthesis of Li_1+γ_V_3_O_8_ via a gel precursor: Part II, from xerogel to the anhydrous material. Chem. Mater. 18, 629–636 (2006).

[b36] Liu L. L. *et al.* Polypyrrole-coated LiV_3_O_8_-nanocomposites with good electrochemical performance as anode material for aqueous rechargeable lithium batteries. J. Power Sources 224, 290–294 (2013).

[b37] Nobili F., Croce F., Scrosati B. & Marassi R. Electronic and electrochemical properties of Li_x_Ni_1-y_Co_y_O_2_ cathodes studied by impedance spectroscopy. Chem. Mater. 13, 1642–1646 (2001).

[b38] Ong C. W., Lin Y. K. & Chen J. S. Effect of various organic precursors on the performance of LiFePO_4_/C composite cathode by coprecipitation method. J. Electrochem. Soc. 154, A527–A533 (2007).

[b39] Wang H., Huang K., Zeng Y., Zhao F. & Chen L. Stabilizing cyclability of an aqueous lithium-ion battery LiNi_1 /3_Mn_1/3_Co_1/3_O_2_/Li_x_V_2_O_5_ by polyaniline coating on the anode. Electrochem. Solid State Lett. 10, A199–A203 (2007).

[b40] Wang H. *et al.* (NH_4_)_0.5_V_2_O_5_ nanobelt with good cycling stability as cathode material for Li-ion battery. J. Power Sources 196, 5645–5650 (2011).

[b41] Feng C., Chew S., Guo Z., Wang J. & Liu H. An investigation of polypyrrole-LiV_3_O_8_ composite cathode materials for lithium-ion batteries. J. Power Sources 174, 1095–1099 (2007).

[b42] Wang H. Y., He H. N., Zhou N., Jin G. H. & Tang Y. G. Electrochemical behavior and cyclic fading mechanism of LiNi_0.5_Mn_0.5_O_2_ electrode in LiNO_3_ electrolyte. Trans. Nonferrous Met. Soc. China 24, 415–422 (2014).

