# Supplementary material for: Advanced aqueous rechargeable lithium battery using nanoparticulate LiTi2(PO4)3/C as a superior anode
Source: Sci Rep. 2015 Jun 2;5:10733. doi: 10.1038/srep10733 (PMC4451790; doi:10.1038/srep10733)
Supplement: Supplementary Information [file srep10733-s1.doc]

**Supporting Information**

Advanced aqueous rechargeable lithium battery using nano- particulate LiTi_2_(PO_4_)_3_/C as a superior anode

Dan Sun^1^, Yifan Jiang^1^, Haiyan Wang*^,1,3,5^, Yan Yao^2^, Guoqing Xu^1^, Kejian He^3^, Suqin Liu^1^, Yougen Tang*^,1,3^, Younian Liu^1^ and Xiaobing Huang^4^

*^1^ College of Chemistry and Chemical Engineering, Central South University, Changsha, 410083,* *P.R China.*

*^2^Department of Electrical and Computer Engineering, University of Houston, Houston, TX 77204, USA*

*^3^Advanced Research Centre, Central South University, Changsha, 410083, P.R. China.*

*^4^College of Chemistry and Chemical Engineering, Hunan University of Arts and Science, Changde, 415000, P.R. China*

*^5^ State Key Laboratory for Powder Metallurgy, Central South University, Changsha 410083, P.R. China*

Tab. S1 The lattice parameters of as-prepared LiTi_2_(PO_4_)_3_/C with different carbon content

| Samples | a/nm | c/nm |
| --- | --- | --- |
| LTP/C-51 | 0.8518 | 2.0898 |
| LTP/C-53 | 0.8519 | 2.1142 |
| LTP/C-55 | 0.8546 | 2.0882 |
| LTP/C-510 | 0.8532 | 2.0753 |

Fig. S1 N_2_ adsorption-desorption isotherm of the LTP/C-55 composite.


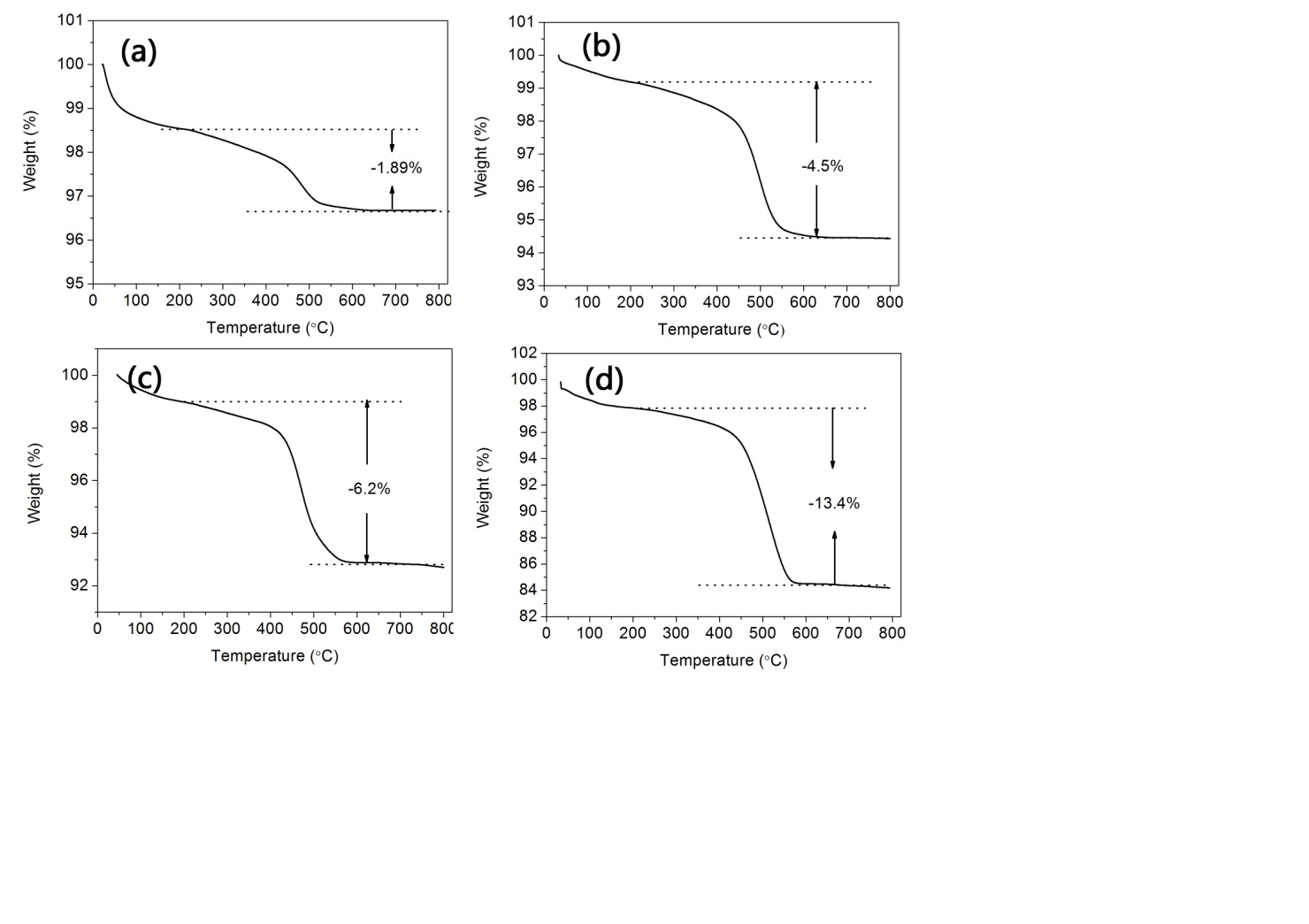


Fig. S2 TG curves of LiTi_2_(PO_4_)_3_/C with different carbon content under airflow: a-LTP/C-51, b-LTP/C-53, c-LTP/C-55, d-LTP/C-510


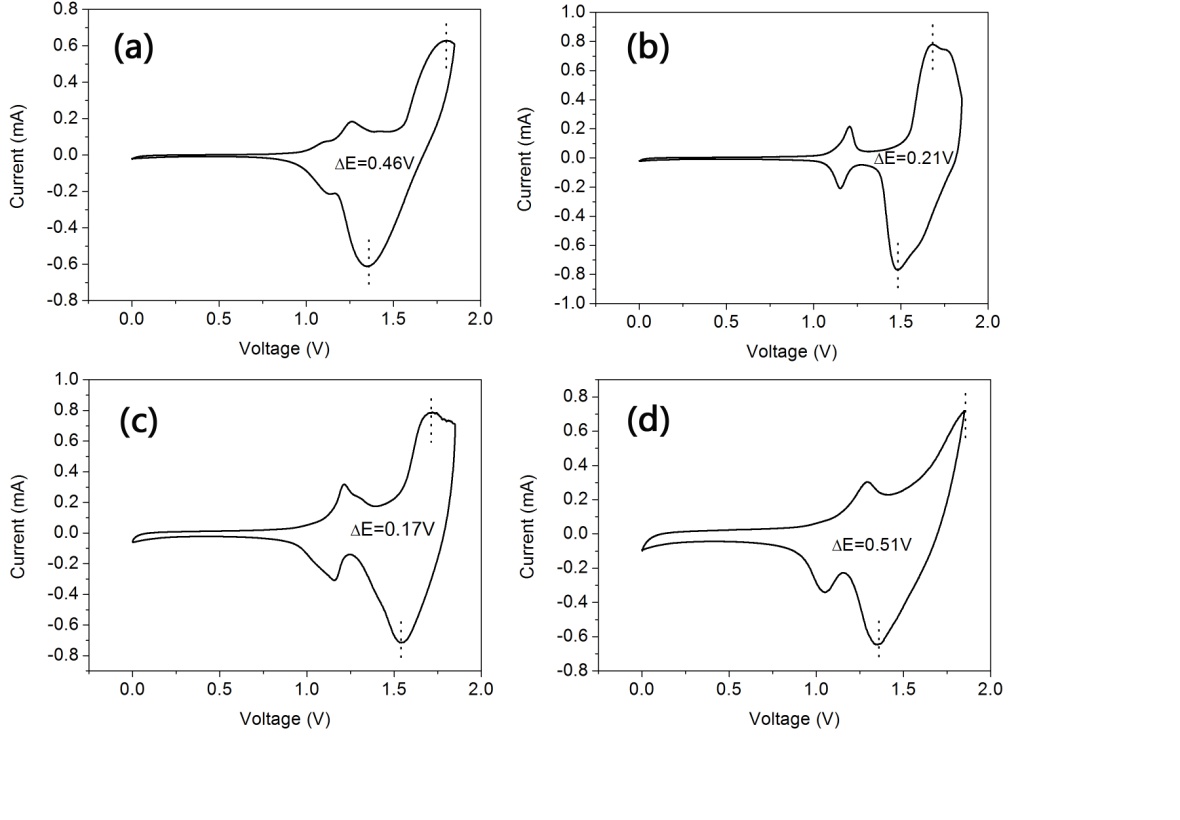


Fig. S3 CV curves of LiTi_2_(PO_4_)_3_/C//LiMn_2_O_4_ ARLB with different LiTi_2_(PO_4_)_3_/C electrodes: a-LTP/C-51, b- LTP/C-53, c- LTP/C-55, d- LTP/C-510.

Tab. S2 Cycling performance of ARLB under different test conditions reported by different research groups

| No. | Battery system | Capacity retention/Cycles | Rate | Ref. |
| --- | --- | --- | --- | --- |
| 1 | VO_2_(B)/C//LiMn_2_O_4_ | Failed/25 | 0.69 mA cm^-2^ | [1](#_ENREF_1) |
| 2 | V_2_O_5_·nH_2_O//LiMn_2_O_4_ | 89%/100 | 50 mA g^-1^ | [2](#_ENREF_2) |
| 3 | H_2_V_3_O_8_^*^ | 72%/50 | 0.1 A g^−1^ | [3](#_ENREF_3) |
| 4 | LiV_3_O_8_//LiMn_2_O_4_ | 78.7%/50 | 0.2 C | [4](#_ENREF_4) |
| 5 | Polyaniline//LiMn_2_O_4_ | 81.4%/150 | 75 mA g^-1^ | [5](#_ENREF_5) |
| 6 | LiV_3_O_8_//LiCo_2_O_4_ | 36%/100 | 3.4 mA cm^-2^ | [6](#_ENREF_6) |
| 7 | LiV_3_O_8_//LiNi_0.81_Co_0.19_O_2_ | 40%/100 | 1 mA cm^-2^ | [7](#_ENREF_7) |
| 8 | TiP_2_O_7_//LiCo_2_O_4_ | 37%/25 | 0.1C | [8](#_ENREF_8) |
| 9 | LiV_3_O_8_^*^ | 30.85%/100 | 0.1C | [9](#_ENREF_9) |
| 10 | LiV_3_O_8_//LiMn_2_O_4_ | 53.5%/100 | 0.2C | [10](#_ENREF_10) |
| 11 | Li_1.2_V_3_O_8_^*^ | 88%/50 | 0.2C | [11](#_ENREF_11) |
| 12 | Polypyrrole-coated LiV_3_O_8_ | 84%/10 | 250 mA cm^-2^ | [12](#_ENREF_12) |
| 13 | Na_2_V_6_O_16_·0.14H_2_O//LiMn_2_O_4_ | 77%/200 | 300 mA g^-1^ | [13](#_ENREF_13) |
| 14 | NaV_6_O_15_//LiMn_2_O_4_ | 80%/400 | 300 mA g^-1^ | [14](#_ENREF_14) |
| 15 | LiTi_2_(PO_4_)_3_/C//LiMn_2_O_4_ | 82%/200 | 10 mA cm^-2^ | [15](#_ENREF_15) |
| 16 | LiTi_2_(PO_4_)_3_/C//LiFePO_4_ | 90%/1000 | 6 C | [16](#_ENREF_16) |
|  |  | 85%/50 | ~0.125C |  |
| 17 | LiTi_2_(PO_4_)_3_/C//LiMn_2_O_4_ | 75%/10 | 0.1C | [8](#_ENREF_8) |
| 18 | LiTi_2_(PO_4_)_3_//LiMn_0.05_Ni_0.05_Fe_0.9_PO_4_ | <80%/50 | 0.2 mA cm^-2^ | [17](#_ENREF_17) |
| 19 | LiTi_2_(PO_4_)_3_/C^*^ | 90%/100 | 0.2C | [18](#_ENREF_18) |
| 20 | LiTi_2_(PO_4_)_3_//LiNi_1/3_Mn_1/3_Co_1/3_O_2_ | 92%/200 | 0.2 mA cm^-2^ | [19](#_ENREF_19) |
| 21 | MoO_3_/PPy// LiMn_2_O_4_ | 90%/150 | 1000 mA g^-1^ | [20](#_ENREF_20) |
| 22 | LiTi_2_(PO_4_)_3_/C//LiMn_2_O_4_ | **90%/300** | **0.2C (30 mA g^-1^)** | This work |
|  |  | **84%/1300** | **1C (150 mA g^-1^)** |  |

* The electrochemical properties of materials are tested using a three-electrode system.

Tab. S3 The lattice parameters of as-prepared LiTi_2_(PO_4_)_3_/C with different sintering time

| Sintering time/hrs | a/ nm | c/ nm |
| --- | --- | --- |
| 5 | 8.5656 | 20.8425 |
| 6 | 8.6161 | 21.2458 |
| 7 | 8.5339 | 20.8482 |

Tab. S4 Nyquist plots of LiTi_2_(PO_4_)_3_/C//LiMn_2_O_4_ ARLB after 5 cycles

| Samples | R_s_ (Ω) | R_ct_ (Ω) |
| --- | --- | --- |
| LTP/C-55 | 1.1 | 39.6 |
| LTP/C-65 | 0.6 | 12.3 |
| LTP/C-75 | 0.6 | 53.8 |

Fig. S4 The charge/discharge curves of bare LTP//LiMn_2_O_4_ ARLB at 0.2C


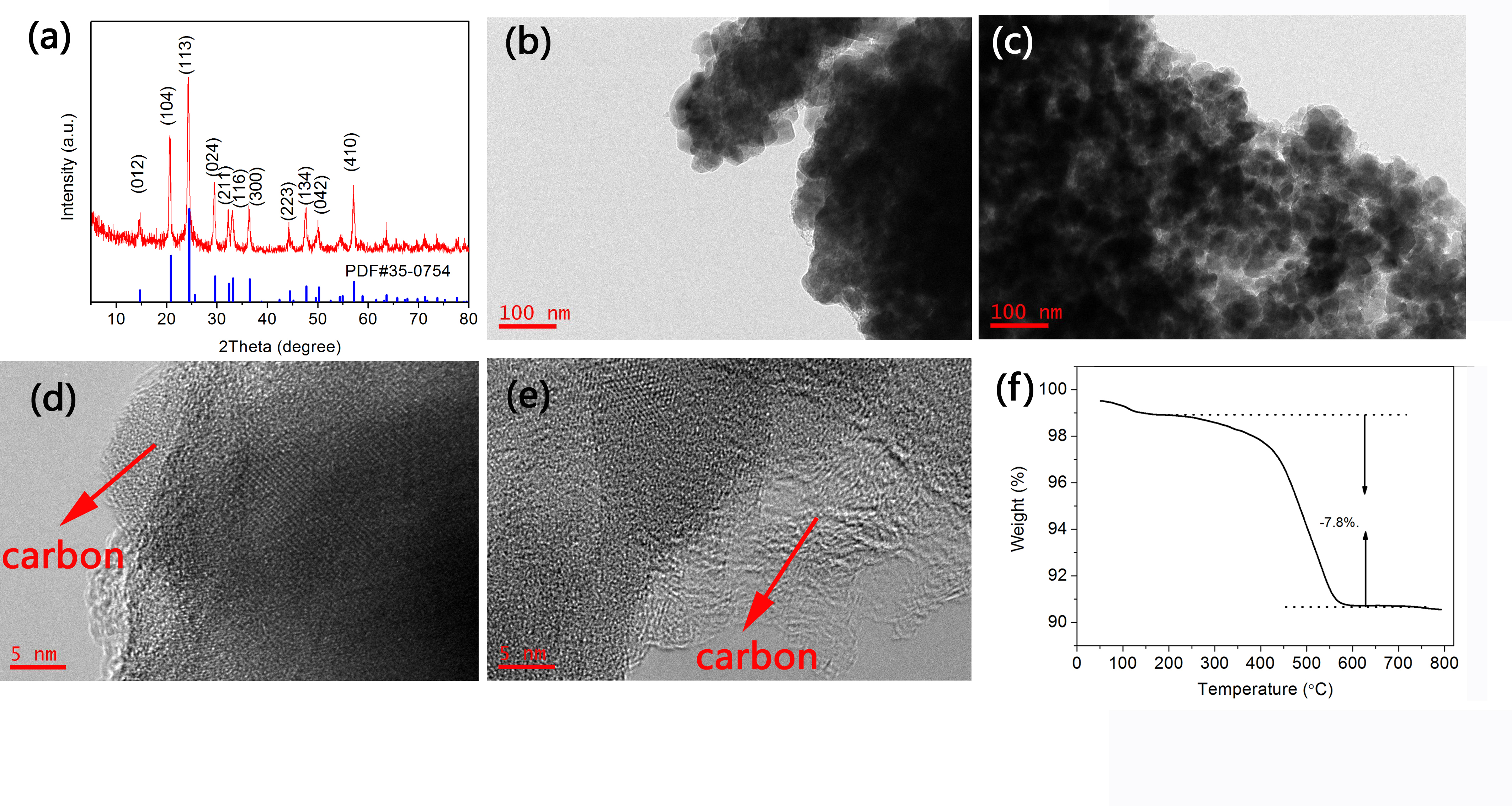


Fig. S5 XRD pattern (a), TEM image (b, c), HRTEM image (d, e) and TG curve (f) of as-prepared LTP/C using sucrose as carbon source.


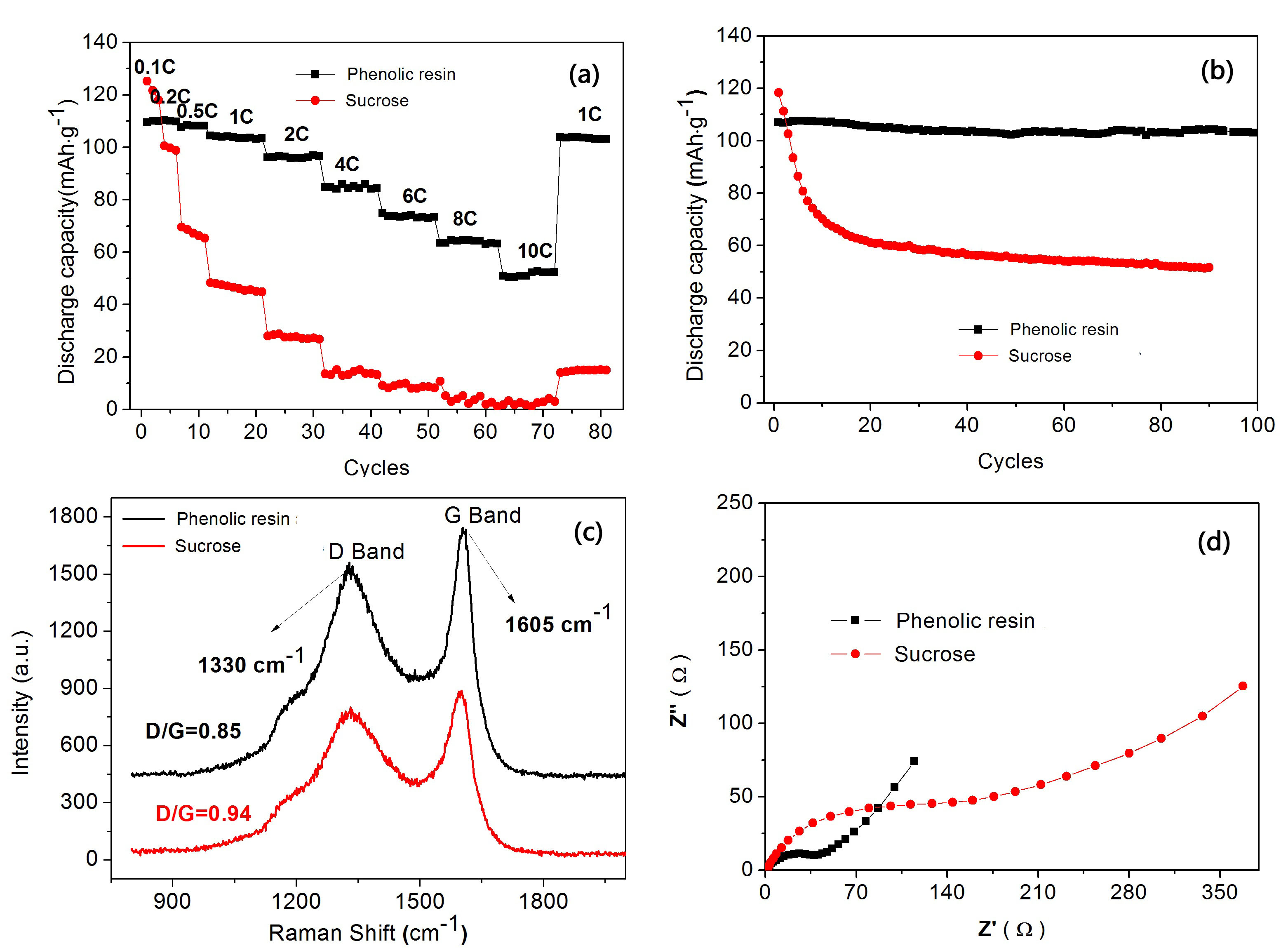


Fig. S6 Comparison of rate performance (a), cycling stability at 1C (b), Raman spectra (c) and EIS spectra (d) of LTP/C with phenolic resin and sucrose as the carbon sources, respectively.

Fig. S7 XRD patterns of LiMn_2_O_4_ electrodes after different cycles.

**References**

1. Li W., Dahn J. R., Wainwright D. S. Rechargeable lithium batteries with aqueous electrolytes. *Science* **264**, 1115-1118 (1994).

2. Stojković I., Cvjetićanin N., Pašti I., Mitrić M., Mentus S. Electrochemical behaviour of V_2_O_5_ xerogel in aqueous LiNO_3_ solution. *Electrochem. Commun.* **11**, 1512-1514 (2009).

3. Li H., Zhai T., He P., Wang Y., Hosono E., Zhou H. Single-crystal H_2_V_3_O_8_ nanowires: a competitive anode with large capacity for aqueous lithium-ion batteries. *J. Mater. Chem.* **21**, 1780-1787 (2011).

4. Zhao M., Zheng Q., Wang F., Dai W., Song X. Electrochemical performance of high specific capacity of lithium-ion cell LiV_3_O_8_//LiMn_2_O_4_ with LiNO_3_ aqueous solution electrolyte. *Electrochim. Acta* **56**, 3781-3784 (2011).

5. Liu L., Tian F., Zhou M., Guo H., Wang X. Aqueous rechargeable lithium battery based on polyaniline and LiMn_2_O_4_ with good cycling performance. *Electrochim. Acta* **70**, 360-364 (2012).

6. Wang G., Fu L., Zhao N., Yang L., Wu Y., Wu H. An aqueous rechargeable lithium battery with good cycling performance. *Angew. Chem. Int. Ed.* **46**, 295-297 (2007).

7. Köhler J., Makihara H., Uegaito H., Inoue H., Toki M. LiV_3_O_8_: characterization as anode material for an aqueous rechargeable Li-ion battery system. *Electrochim. Acta* **46**, 59-65 (2000).

8. Wang H., Huang K., Zeng Y., Yang S., Chen L.. Electrochemical properties of TiP_2_O_7_ and LiTi_2_(PO_4_)_3_ as anode material for lithium ion battery with aqueous solution electrolyte. *Electrochim. Acta* **52**, 3280-3285 (2007).

9. Liu L., Tian F., Yang Z., Wang X., Zhou M., Wang X. Electrochemical behavior of nanostructured LiV_3_O_8_ in aqueous LiNO_3_ solution. *J. Phys.Chem. Solids* **72**, 1495-1500 (2011).

10. Wang G. J., Zhang H. P., Fu L. J., Wang B., Wu Y. P. Aqueous rechargeable lithium battery (ARLB) based on LiV_3_O_8_ and LiMn_2_O_4_ with good cycling performance. *Electrochem. Commun.* **9**, 1873-1876 (2007).

11. Stojkovic I., Cvjeticanin N., Mitric M., Mentus S. Electrochemical properties of nanostructured Li_1.2_V_3_O_8_ in aqueous LiNO_3_ solution. *Electrochim. Acta* **56**, 6469-6473 (2011).

12. Liu L. L., Wang X. J., Zhu Y. S., Hu C. L., Wu Y. P., Holze R. Polypyrrole-coated LiV_3_O_8_-nanocomposites with good electrochemical performance as anode material for aqueous rechargeable lithium batteries. *J. Power Sources* **224**, 290-294 (2013).

13. Zhou D., Liu S., Wang H., Yan G. Na_2_V_6_O_16_·0.14H_2_O nanowires as a novel anode material for aqueous rechargeable lithium battery with good cycling performance. *J. Power Sources* **227**, 111-117 (2013).

14. Sun D*, et al.* Aqueous rechargeable lithium batteries using NaV_6_O_15_ nanoflakes as high performance anodes. *J. Mater. Chem. A* 2, 12999-13005 (2014).

15. Luo J. Y., Xia Y. Y. Aqueous lithium-ion battery LiTi_2_(PO_4_)_3_/LiMn_2_O_4_ with high power and energy densities as well as superior cycling stability. *Adv. Funct. Mater.* **17**, 3877-3884 (2007).

16. Luo J. Y., Cui W. J., He P., Xia Y. Y. Raising the cycling stability of aqueous lithium-ion batteries by eliminating oxygen in the electrolyte. *Nat.Chem.* **2**, 760-765 (2010).

17. Liu X. H., Saito T., Doi T., Okada S., Yamaki J. I. Electrochemical properties of rechargeable aqueous lithium ion batteries with an olivine-type cathode and a Nasicon-type anode. *J. Power Sources* **189**, 706-710 (2009).

18. Wessells C., La Mantia F., Deshazer H., Huggins R. A., Cui Y. Synthesis and electrochemical performance of a lithium titanium phosphate anode for aqueous lithium-ion batteries. *J. Electrochem. Soc.* **158**, A352-A355 (2011).

19. Shivashankaraiah R. B., Manjunatha H., Mahesh K. C., Suresh G. S., Venkatesha T. V. Electrochemical characterization of LiTi_2_(PO_4_)_3_ as anode material for aqueous rechargeable lithium batteries. *J. Electrochem. Soc.* **159**, A1074-A1082 (2012).

20. Tang W., Liu L., Zhu Y., Sun H., Wu Y., Zhu K. An aqueous rechargeable lithium battery of excellent rate capability based on a nanocomposite of MoO_3_ coated with PPy and LiMn_2_O_4_. *Energy Environ. Sci.* **5**, 6909-6913 (2012).
